# Nanowire Quantum Dot Emitters Evanescently Coupled On-Chip via Curved and Segmented SiN Waveguides

**DOI:** 10.1021/acsphotonics.6c00397

**Published:** 2026-07-24

**Authors:** Edith Yeung, Kataryna Sorensen, David B. Northeast, Maziyar Milanizadeh, Philip J. Poole, Robin L. Williams, Dan Dalacu

**Affiliations:** † University of Ottawa, Ottawa, Ontario K1N 6N5, Canada; ‡ 6356 National Research Council Canada, Ottawa, Ontario K1A 0R6, Canada

**Keywords:** nanowires, quantum, dots, photons, sources

## Abstract

This work implements a hybrid device based on a semiconductor quantum dot embedded within a nanowire to bridge a noncontinuous curved waveguide structure. The geometry takes advantage of evanescent coupling between the photonic structures to recover single photons emitted from both outputs of the device. Auto- and cross-correlation measurements were performed on different output facets of the device. We demonstrate single-photon emission from both ends of the nanowire for both neutral, *X* and *XX*, and charged *X* ^–^, excitonic complexes. We further demonstrate the cascaded *XX* – *X* emission by collecting each complex from a different facet. This work lays the foundation for on-chip architectures which utilize multidirectional integration of quantum emitters.

## Graphical abstract

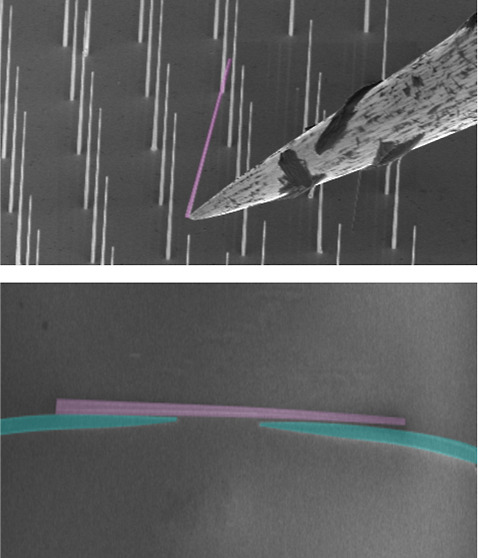

## Introduction

Sources of single photons are a key resource in quantum information processing applications,^1^ and solid-state two-level systems^2^ offer a promising route to generating single-photons on-demand with high efficiency. Semiconductor quantum dots (QDs) are of particular relevance as they have achieved nearly ideal performance when integrated with photonic structures.^3–9^ These structures, however, are typically designed to operate out-of-plane, whereas future applications will likely require the scalability provided by on-chip integration.^10–13^

QDs embedded within nanowires that are grown^14^ (i.e., bottom-up) as opposed to etched^15^ (i.e., top-down) provide a route to fabricating sources of single photons deterministically. The number of emitters per device is controlled, and each device is grown at a specified position on the substrate. Bottom-up nanowire QDs have been used to generate both high-quality single photons^16,17^ and high-fidelity entangled photon pairs.^18,19^ These sources were also operated out-of-plane, and high efficiency was obtained by designing the nanowire to act as a single-mode waveguide supporting only the fundamental HE_11_ mode,^20,21^ and by introducing a taper along the length of the nanowire^22^ to expand the mode for efficient coupling to external optics. Properly designed, 95% of the dot emission is directed into the HE_11_ mode,^21^ which can be coupled to single-mode fiber with an efficiency of 93%.^23^

The ability to control the nanowire taper is also relevant for designing chip-integrated sources.^24,25^ In this case, the mode expansion provided by the taper is not for efficient coupling to low numerical aperture optics but rather for efficient evanescent coupling to on-chip waveguides. By placing an appropriately tapered nanowire along a straight Si_3_N_4_ waveguide, as in Figure 1a, the HE_11_ mode can be efficiently coupled on-chip, with simulated coupling efficiencies approaching 100%.^24,25^ The hybrid structures can be assembled by picking up nanowires from the growth substrate and placing them on Si_3_N_4_ waveguides using a scanning electron microscope (SEM)-based nanomanipulator.^24,26^ With this approach, we have demonstrated >90% coupling of the HE_11_ mode to a Si_3_N_4_ waveguide and have generated on-chip single photons with purities exceeding 95%.^25^

**Figure 1:**
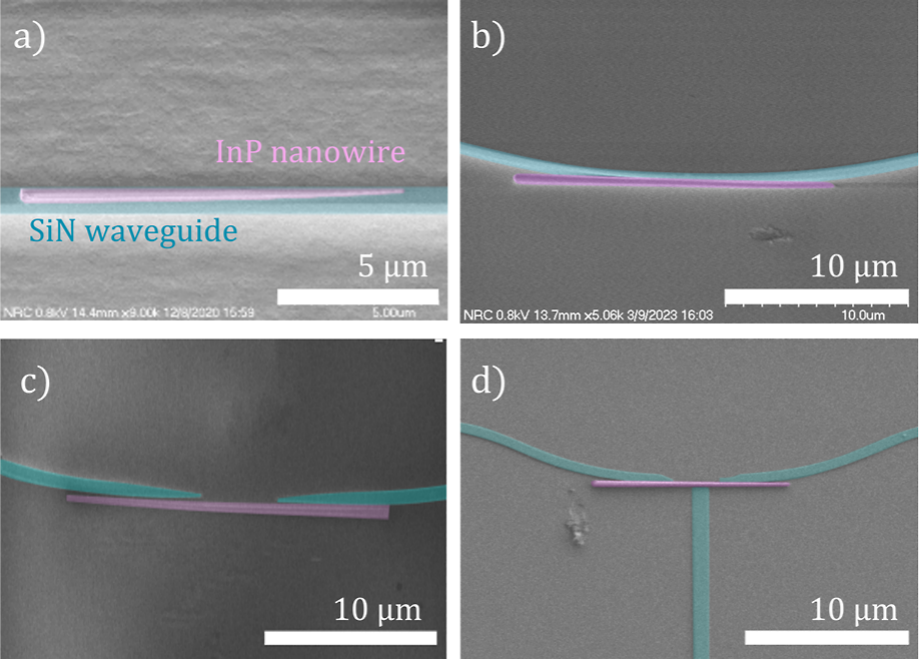
False color SEM images with SiO_2_ in gray, Si_3_N_4_ in blue, and InP in purple. (a) Straight Si_3_N_4_ waveguide with a tapered InP nanowire on top. (b) Curved waveguide with a nanowire on the side. (c) Segmented waveguide with a nanowire bridging the gap. (d) As in (c) but with an additional waveguide for cross-pumping.

The pick-and-place hybrid platform is not limited to simple structures such as that shown in Figure 1a: one can also construct hybrid devices consisting of a nominally untapered nanowire with a thinner diameter placed beside a curved waveguide, as shown in Figure 1b. In this case, the QD within the nanowire is located at its midpoint and the evanescent transfer of the nanowire mode to the waveguide occurs as the separation between the two increases. Such a device can harvest photons emitted in either direction along the nanowire waveguide, a limitation of the unidirectional structure in Figure 1a where emission toward the base is not efficiently collected.

Expanding on the curved waveguide architecture, a gap can be introduced in the waveguide with the nanowire connecting the circuit, as shown in Figure 1c. In this case, the waveguide mode can be transferred completely to the nanowire and back again, assisted by tapering each end of the segmented waveguide. This structure is relevant for new types of experiments such as transmission measurements to investigate coherent interactions between single photons and two-level systems.^27^ By incorporating an additional waveguide orthogonal to the nanowire, as in Figure 1d, we can also envision a resonant fluorescent excitation scheme where pump laser rejection is achieved using orthogonal excitation and collection directions.^28–30^

In this article, we explore some of the advanced architectures shown in Figure 1, specifically (b) and (c), for on-chip single-photon generation in Si-based integrated photonic circuitry, i.e., Si_3_N_4_ waveguides. We evaluate, through simulations, geometry-dependent coupling efficiencies between a nanowire and a curved waveguide both with and without a gap. We find the segmented waveguide design more robust against variations in the nanowire diameter when collecting from curved waveguide geometries and use this architecture to demonstrate efficient generation of on-chip high-purity single photons. We further utilize the bidirectional nature of the device to perform cross-correlation measurements by collecting from both ends of the nanowire with the nanowire acting as an integrated beamsplitter.

### Simulations

As mentioned in the Introduction section, a nanowire containing a quantum emitter can transfer its light efficiently to a nearby waveguide by evanescent coupling.^24,25^ This process is robust provided both structures allow for a single mode and a tapered section is included over a sufficient length to allow adiabatic transfer of mode energy between the two. In this scenario, light that is emitted away from the taper—50% of light from the QD—will encounter an abrupt end of the nanowire and will couple poorly into the waveguide. A waveguide that curves as it approaches the nanowire will be seen as a gradual change in the refractive index. Device designs can use this effect to allow for light coupling in both directions. Since QD emission has a narrow bandwidth and optical response of all structures is expected to change slowly with wavelength, frequency domain finite element simulations (COMSOL Multiphysics) are performed to predict device performance.

We first investigate power transfer from a dipole in a straight InP nanowire to a curved Si_3_N_4_ waveguide. A 20 μm nanowire length is used in order to provide sufficient separation between the nanowire ends and the curved waveguide for a waveguide with a 30 μm radius of curvature. The Si_3_N_4_ waveguides are 0.4 μm thick and 0.5 μm wide, chosen to support a single mode in both TE and TM polarizations, sitting on 4 μm of SiO_2_. The nanowire, with radius *r*_nw_, is placed as seen in Figure 2a such that the dipole is located precisely where the nanowire meets the waveguide. The figure shows the electric field polarized along the TE direction of the waveguide. In the inset, we show the fraction of the total power radiated from the dipole that couples to the waveguide for different *r*_nw_. We calculate a peak coupling of greater than 76% in both waveguide directions which drops quickly for nanowire radii away from the optimal value of *r*_nw_ = 115 nm.

**Figure 2:**
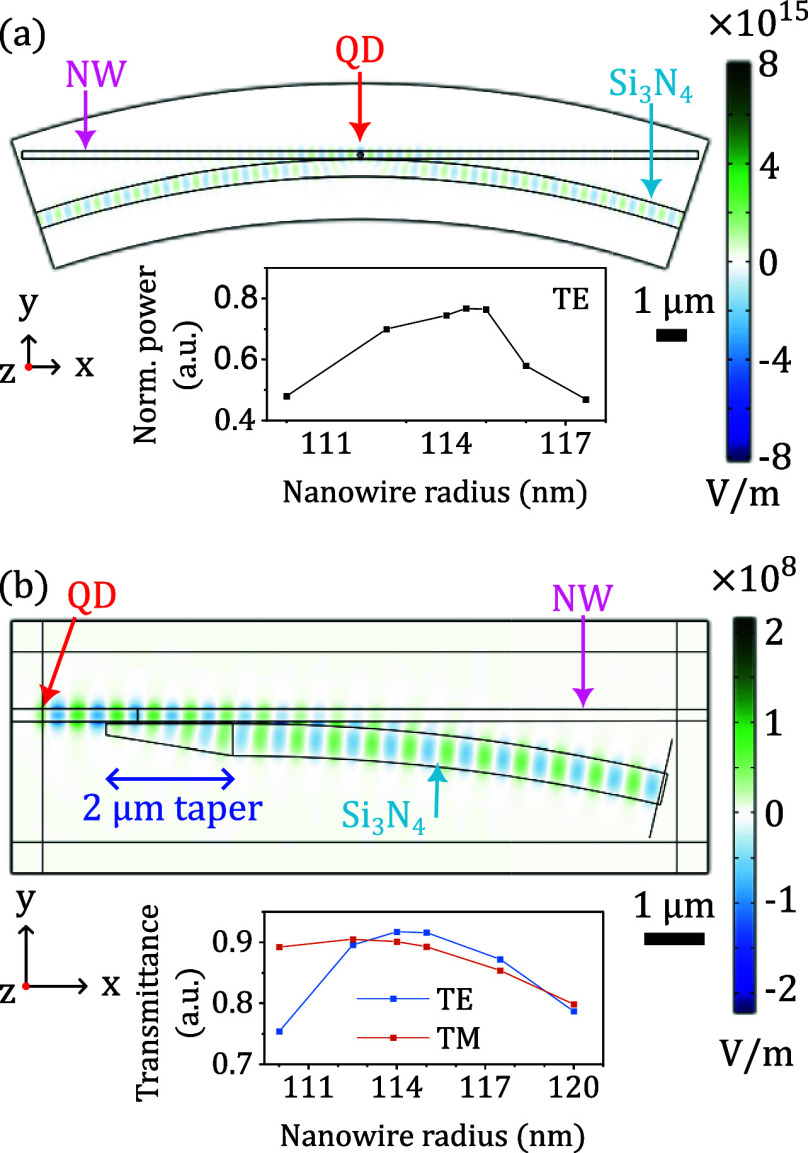
Finite element simulations were performed for the two different hybrid coupling approaches. (a) Top-down view of the photonic integrated circuit. The QD emitter (here a dipole) is located at the midpoint of the InP nanowire (NW) which is tangent to the curved Si_3_N_4_ waveguide. The emission from the QD is emitted into the nanowire and couples into the waveguide as it bends away. The total power coupled into the waveguide as a function of nanowire radius, *r*_nw_, is shown in the inset. (b) Revised coupling design that makes use of a gap in the Si_3_N_4_ waveguide with tapered sections at the gap. The inset shows the improved transmittance between both HE_11_ polarizations in the nanowire and the TE/TM modes in the Si_3_N_4_ ridge waveguide. Electric field simulations in both (a) and (b) are shown for optimal nanowire radii.

Combining the curved geometry with a tapered waveguide, power transfer can be made less sensitive to the nanowire radius, *r*_nw_. Figure 2b depicts a curved Si_3_N_4_ waveguide that tapers to an end over a length of 2 μm. Simulated transmittance of HE_11_ nanowire modes to TE and TM waveguide modes is shown in the inset for different *r*_nw_. We calculate a peak transmission of over 90% for both linear polarizations and observe a reduced dependence on *r*_nw_ compared to the untapered waveguide geometry in Figure 2a.

In summary, these simulations show that curved waveguide geometries allow for the efficient collection of QD emission directed toward either end of the InP nanowire. This is in contrast to tapered nanowires on straight waveguides where coupling efficiency for emission toward the base of the nanowire is low and scattering at the base is high.^24,25^ When comparing the continuous curved waveguide with the segmented waveguide, the simulations show that the segmented tapered Si_3_N_4_ geometry provides higher coupling values with a higher tolerance for variations in nanowire radius. In the experiments described below, we will focus on the segmented tapered Si_3_N_4_ geometry.

### Experimental Details

The quantum emitters used in this work are InAsP QDs embedded within InP nanowires. An array of position-controlled InP nanowires are grown using selective-area vapor–liquid–solid epitaxy, see refs 14,16,17 for details. In the first growth stage, we grow a single InAsP QD as a segment within a 20 nm diameter InP nanowire core at a nominal height of 10 μm above the substrate. In the second growth stage, the nanowire core is clad with InP to increase the diameter to 210 nm and to increase the nanowire height to 20 μm such that the QD is at the midpoint of the nanowire. In this cladding stage, growth conditions are adjusted to minimize tapering of the nanowire diameter along its length as the proposed designed is based on an untapered nanowire, see Figure 2.

The integrated photonic circuitry is based on low-pressure chemical vapor-deposited Si_3_N_4_ waveguides fabricated on Si wafers using the commercial foundry Ligentec. Waveguide dimensions are 0.4 μm tall and 0.5 μm wide clad with 3.3 μm of SiO_2_ above and 4 μm below. For coupling on and off chip, we use edge couplers designed for lensed fibers: at the etched facets, the waveguide widths are tapered to 0.2 μm over a length of 150 μm. The segmented waveguide consists of a curved section with a 30 μm radius of curvature and with a 4 μm gap at the midpoint. On either side of the gap, the waveguide is tapered down to 0.180 μm over 2.0 μm, as shown in Figure 2b. A window in the top SiO_2_ layer is opened to expose just the segmented waveguide section, permitting nanowire placement. The etch depth of the SiO_2_ exposing part of the Si_3_N_4_ waveguide was chosen to allow the nanowire to sit roughly at the midpoint (in height) of the waveguide. We note that the asymmetry in the *z*-direction within the window defines the two orthogonally polarized HE_11_ nanowire modes: one along z and the other parallel to the substrate, i.e., corresponding to the TE and TM modes, respectively, of the Si_3_N_4_ waveguide.

The precharacterized nanowire is picked up from the growth substrate with a piezomotor-controlled tungsten tip on the nanomanipulator inside an SEM.^24,25^ The nanowire is placed tangentially to the curved waveguide such that it bridges the gap with the midpoint of the nanowire, and hence the QD, centered in the gap. An example of the final structure, which we term a gap-coupled device, is shown in Figure 1c.

The device was measured in a fiber-coupled closed-cycle helium cryostat at 5.5 K. The cryostat is equipped with three *xyz* piezo stacks: one for free-space alignment and two for aligning fibers to edge facet couplers. We take advantage of the on-chip platform to pump the QD and collect the emission in three different configurations: (*i*) pump via fiber through the waveguide from one side and collect from the other side, (*ii*) pump and collect from the same side using a 90:10 fiber splitter (here we can also simultaneously collect from the other side), and (*iii*) pump from the top using free-space optics and collect from both sides of the segmented waveguide. For coupling off- and on-chip, we used PM 780HP lensed fibers with a mode field diameter of 5.3 ± 0.1 μm at 850 nm. For free-space pumping and for imaging, a 100× objective (NA = 0.81) placed inside the cryostat was used.

Photoluminescence (PL) measurements were done using above-band continuous wave or pulsed laser excitation. The emission was collected using one of the above configurations and sent to a fiber-coupled grating spectrometer for spectrally resolved measurements or to a fiber-coupled narrow-band (bandwidth *BW* = 0.1 nm) tunable filter for measurements on single excitonic lines. For measurements on the single excitonic complexes, including time-resolved PL, high-resolution PL, and time-correlated PL, emitted photons were detected using superconducting nanowire single-photon detectors (SNSPDs) with efficiencies of 80% and timing jitters of 100 ps.

## Results

In Figure 3, we show a PL spectrum from a gap-coupled device, e.g., Figure 1c. The measurement was performed by exciting through the waveguide from one side and collecting from the other side. The spectrum shows three dominant peaks, typical in this QD system (see, for example, ref 31). The peaks are identified as emission from the neutral exciton (*X*), biexciton (*XX*), and trion (*X* ^–^) where peak identification is based on the intensity dependence on pump power (see inset) as well as high-resolution PL and cross-correlation measurements discussed below.

**Figure 3:**
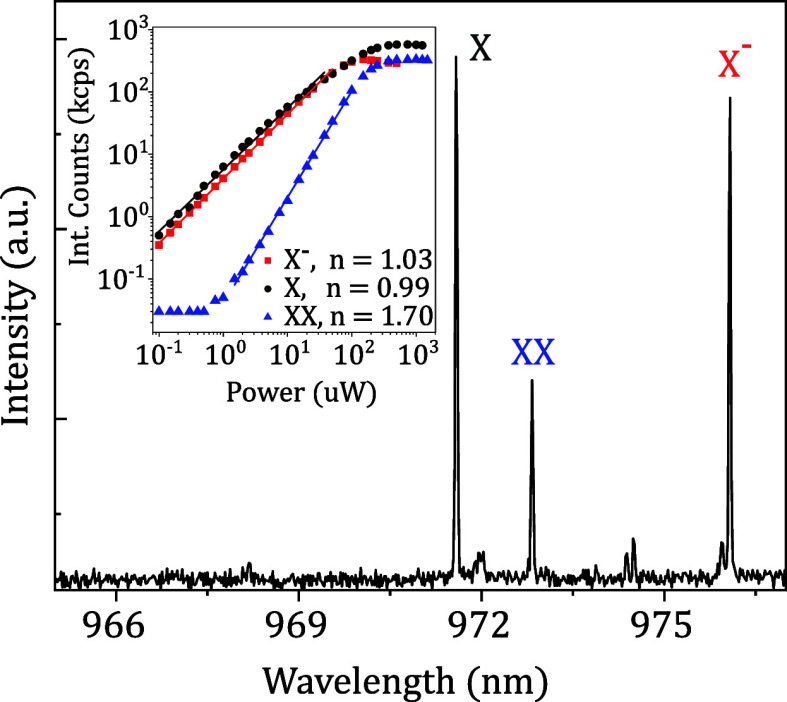
PL spectrum measured on a gap-coupled device showing three dominant peaks identified as the excitonic complexes *X*, *XX*, and *X* ^–^. The spectrum was taken with above-band (λ = 780 nm) pulsed excitation at 80 MHz using an excitation power of 150 μW (*P* = 0.7*P*_sat_). The inset shows the integrated intensity as a function of power for each complex: linear (*n*–1) for *X* (black circles) and *X* ^–^ (red squares) and approaching quadratic (*n*–2) for *XX* (blue triangles).^32^

The integrated intensities from each complex for pulsed excitation at 80 MHz at saturation, *P*_sat_, were 575 kcps for *X*, 330 kcps for *XX*, and 330 kcps from *X* ^–^. Using these values, we obtain end-to-end efficiencies (detected counts/80 MHz) of 0.72%, 0.41%, and 0.41% for *X*, *XX*, and *X* ^–^, respectively. These efficiencies are limited predominantly by the throughput of the setup (<10%) and by the loss of 50% of the photons directed toward the pump direction as here we are only collecting from one side. Additional losses include the nanowire to waveguide coupling, waveguide propagation loss, facet coupling, and the detector efficiency, see the Supporting Information for a detailed loss analysis.

If we reverse the excitation and collection waveguides, we observe a decrease in the count rate of 2 orders of magnitude, whereas simulations predict equal coupling in both directions. This asymmetry may be due to a displacement of the QD from the center of the gap in the waveguide, either from the dot not centered in the InP nanowire and/or the nanowire not centered in the gap, see the Supporting Information. A direction-dependent coupling may also arise from an unintentional taper of the nanowire that can be observed in Figure 1c. Nonetheless, we obtain sufficient counts from both directions to perform the bidirectional measurements that will be discussed below.

Time-resolved PL traces of the three complexes are shown in Figure 4b,d,f where we excite from one side and collect for the other as above, see Figure 4a. To extract decay rates, we assume a double exponential of the form^33^

1 $$I\left(\tau \right)=A_{b}e^{-\tau /\tau_{b}}+A_{d}e^{-\tau /\tau_{d}}+y_{0}$$

where τ is the delay between a synchronization pulse from the laser and a detection event, 1/τ_*b*_ is the radiative decay rate of the bright exciton, 1/τ_*d*_ is the spin-flip rate that converts the system from a dark to bright state with *A*_*b*_ and *A*_*d*_ the amplitudes of the bright and dark contributions, respectively, and *y*_0_ is a background offset. The ratio *A*_*b*_/*A*_*d*_ is a measure of the occupation probability of the bright state. Model fits to the decay curves are shown as red lines in Figure 4b,d,f, and extracted parameters τ_*b*_, τ_*d*_ and *A*_*b*_/*A*_*d*_ are summarized in Table 1 for all three complexes.

**Figure 4:**
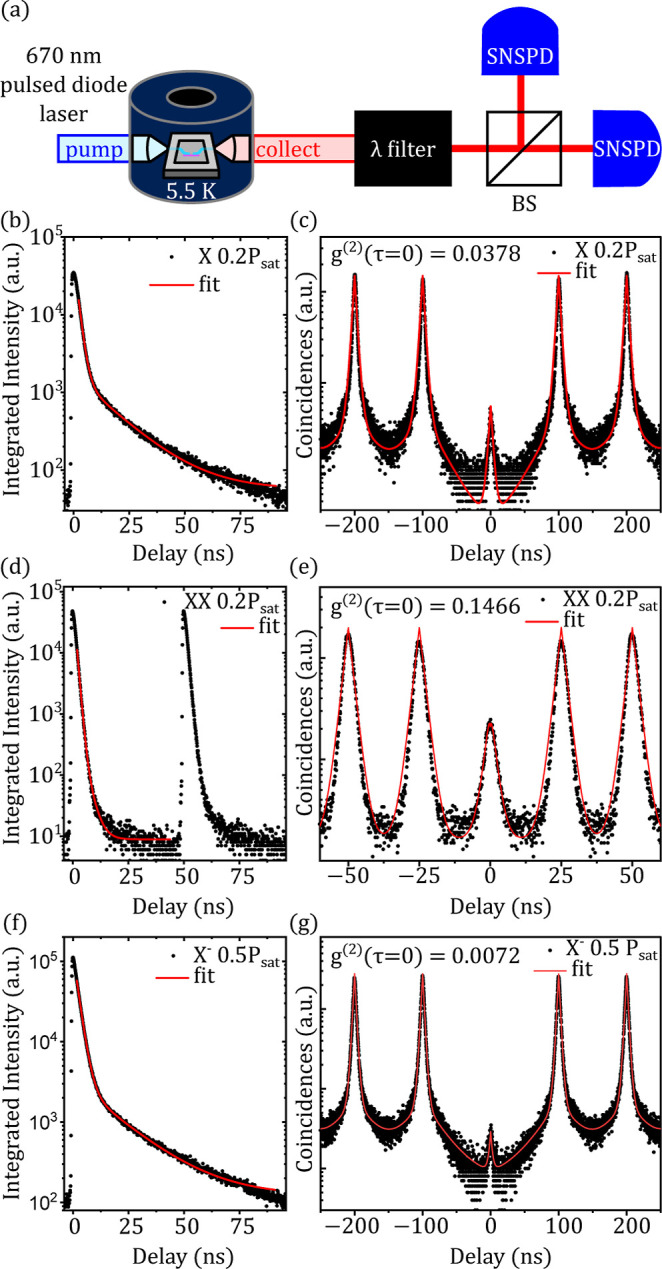
(a) Schematic of the setup for measuring *g* ^(2)^(τ). The excitonic complex to be measured is first filtered then directed to a beamsplitter with the output ports sent to two SNSPD detectors. (b, d, f) PL decay traces and (c, e, g) autocorrelation measurement for the three complexes, *X*, *XX*, and *X* ^–^, respectively, plotted semilogarithmically. The excitation rate used in each measurement is shown in the figure as a fraction of the saturation power, *P*_sat_.

**Table 1:** Summary of Parameters Extracted from Model Fits to the PL Decay Traces and Second-Order Correlation Measurements

complex	τ_*b*_ (ns)	τ_*d*_ (ns)	*A*_*d*_/*A*_*b*_	*g* ^(2)^(τ = 0)
*X*	1.680 ± 0.004	16.2 ± 0.2	0.0296	0.03776
*XX*	0.982 ± 0.006	2.8 ± 0.3	0.011	0.14663
*X* ^–^	2.107 ± 0.002	18.2 ± 0.2	0.046	0.00724

The time-resolved PL traces of the *X* and *X* ^–^ emission, Figure 4b,f, respectively, both show strong slow decay contributions with decay times, τ_*d*_, consistent with spin-flip rates of neutral excitons in QD systems.^33^ For *X* ^–^, the spin-flip is associated with a carrier in a p-shell of the dot: a dark excited state of the complex that relaxes to the bright state.^17^ In contrast, the PL decay of the *XX* emission, Figure 4d, is predominantly monoexponential with a possible small contribution from a slightly slower process.

Second-order autocorrelations, *g* ^(2)^(τ), measured using pulsed diode excitation (λ = 670 nm, pulse width = 100 ps) in a Hanbury-Brown and Twiss (HBT) setup, see Figure 4a, are shown in Figure 4c,e,g for *X*, *XX*, and *X* ^–^, respectively. Due to the significant slow component in the decay of *X* and *X* ^–^, an excitation repetition rate of 10 MHz was used to allow the system to fully decay between pulses. For *XX*, where τ_*d*_ was significantly shorter, the dot was excited at 40 MHz. The reduced zero-delay peak in the *g* ^(2)^(τ) for all three complexes demonstrates single-photon emission. To quantify the single-photon purity, the *g* ^(2)^(τ) is modeled by

2 $$\begin{array}{ll} g^{\left(2\right)}\left(\tau \right)= & C_{0}\left[e^{-\left|\tau \right|/\tau_{b}}+\frac{A_{d}}{A_{b}}e^{-\left|\tau \right|/\tau_{d}}\right]-B_{0}e^{-\left|\tau \right|/\tau_{re}}\\ & +C_{1}\sum_{n\neq 0}\left[e^{-\left|\tau \right|-nt_{rep}/\tau_{b}}+\frac{A_{d}}{A_{b}}e^{-\left|\tau \right|-nt_{rep}/\tau_{d}}\right]\\ & +C_{b} \end{array}$$

where *C*_0_ is the amplitude of the coincidences due to the multiphoton emission events at τ = 0, *B*_0_ is associated with re-excitation of the dot, at a rate of 1/τ_re_, from the same excitation pulse, *C*_1_ corresponds to coincidences recorded from different excitation pulses at τ = *nt*_rep_ for *n* ≠ 0, and *C*_*b*_ represents the background counts. For the remaining parameters, values extracted from the fits to the time-resolved PL are used. The model fits are shown in Figure 4c, e, g (red curves) for the three complexes. From the ratio of the area of the zero delay peak to the average peak area at τ = *nt*_rep_, we calculate the probability of multiphoton emission, *g* ^(2)^(τ = 0), listed in Table 1.

We obtain very low probabilities of multiphoton emission from *X* and *X* ^–^ with *g* ^(2)^(τ = 0) values of <4% and <1%, respectively. Coincidences in the zero-delay peak are associated with re-excitation of the dot from the same laser pulse, previously seen in this system with above-band excitation.^34–36^ We note that the dip in the zero-delay peak that is characteristic of re-excitation is not observed and we speculate that the time scales associated with the re-excitation process are too fast to be resolved using our 100 ps detectors. The higher *g* ^(2)^(τ = 0) for *XX* of nearly 15% may be related to the faster decay (τ_*b*_ = 982 ps) and/or higher excitation power required for saturation, both of which would lead to an increased re-excitation probability during the 100 ps excitation pulse.

To evaluate the spectral purity of the emitted photons, we measure high-resolution PL spectra for each complex by directing the filtered emission through a tunable Fabry–Perot etalon with a bandwidth of *BW* = 200 MHz, sufficiently narrow to resolve real line shapes. High-resolution spectra of *X* and *XX* are shown in Figure 5a,b, respectively. In each case, two peaks are resolved corresponding to the two orthogonal exciton states separated by the fine structure spitting, FSS.^37^ The peaks are fit with Voigt line shapes from which we extract an FSS = 2.55 ± 0.02 GHz for *X* and 2.96 ± 0.01 GHz for *XX*. These values of FSS are larger than we typically observe in this system^18^ which we associated with slower switching between Group V precursors during the dot growth in this sample that results in an axial asymmetry due to P and As tailing.^38^

**Figure 5:**
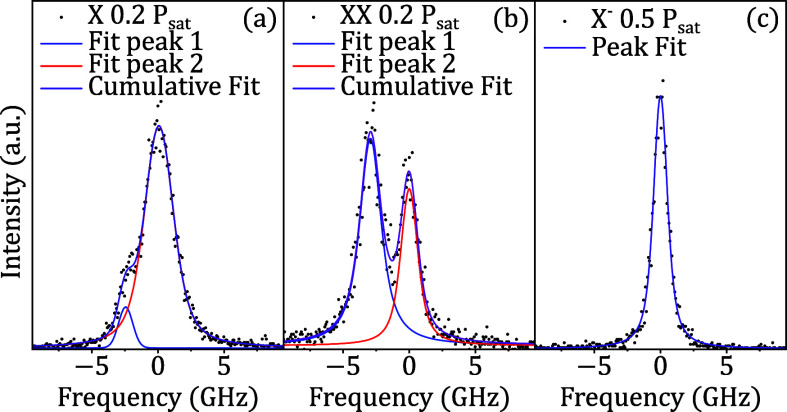
High-resolution PL of the (a) *X*, (b) *XX*, and (c) *X* ^–^ peaks measured at excitation powers indicated. Model fits are shown in purple. For the neutral complexes, the two peaks that constitute the doublets are shown in blue and red.

The extracted line widths are $\delta \omega_{V,X_{1}}=1.83\pm 0.07\;GHz$ and $\delta \omega_{V,X_{2}}=2.59\pm 0.03$ GHz for the two *X* dipoles and $\delta \omega_{V,XX_{1}}=1.85\pm 0.05$ GHz and $\delta \omega_{V,XX_{2}}=1.66\pm 0.05$ GHz for the two *XX* dipoles. In an ideal system, the homogeneous broadening of the line width is limited by its lifetime giving a Lorentzian line shape with a transform-limited line width of δω = 1/2πτ_b_.^39^ We observe Voigt line shapes indicating a Gaussian component (i.e., inhomogeneous broadening) likely from a fluctuating charge environment^40^ common with above-band excitation. Using measured lifetimes, we calculate transform-limited line widths of 1/2πτ_b,*X*_ = 0.0947 GHz for *X* and 1/2πτ_b,*XX*_ = 0.162 GHz for *XX*. Observed line widths are 19.3 and 27.3 (11.4 and 10.2) times this limit for *X* (*XX*), depending on the dipole, which gives a measure of the charge noise. The reduced excess broadening observed for *XX* may indicate a reduced sensitivity to charge fluctuations for complexes with beginning and end states both modified by the presence of charge; for *X*, where the final state in empty, this is not the case.

For the *X* ^–^ transition, shown in Figure 5c, a single peak is observed, consistent with its identification as a singly charged complex and thus not expected to have a *FSS*.^37^ The line shape here is also Voigt with a line width of $\delta \omega_{V,X^{-}}=1.311\pm 0.007$ GHz, 17.4 times the transform-limited line width of $1/2\pi \tau_{b,X^{-}}=0.0755$ GHz. We note that above-band excitation is not expected to produce lifetime-limited photons,^41^ however, the nanowire QD system has been shown to approach this limit.^39^ It is possible that slower Group V switching in this sample may have resulted in more pronounced compositional tails,^38^ effectively increasing the dot thickness and making it more susceptible to fluctuations in the charge environment.

Measurements thus far have demonstrated efficient coupling of single photons on chip, similar to previous experiments using straight waveguides.^24,25^ Next we harness the versatility of the gap-coupled architecture to perform bidirectional measurements. In this case, we excite the nanowire from above using free-space optics and collect from both edge facets, as shown in Figure 6a. Figure 6b compares time-resolved PL traces of the *X* ^–^ emission for collection from the left and right facets. The two traces lie on top of each other with decay times equivalent to that obtained when excitation was via a waveguide, see Figure 4f. This measurement simply confirms that the excitation and collection configuration does not impact the decay processes of the transition.

**Figure 6:**
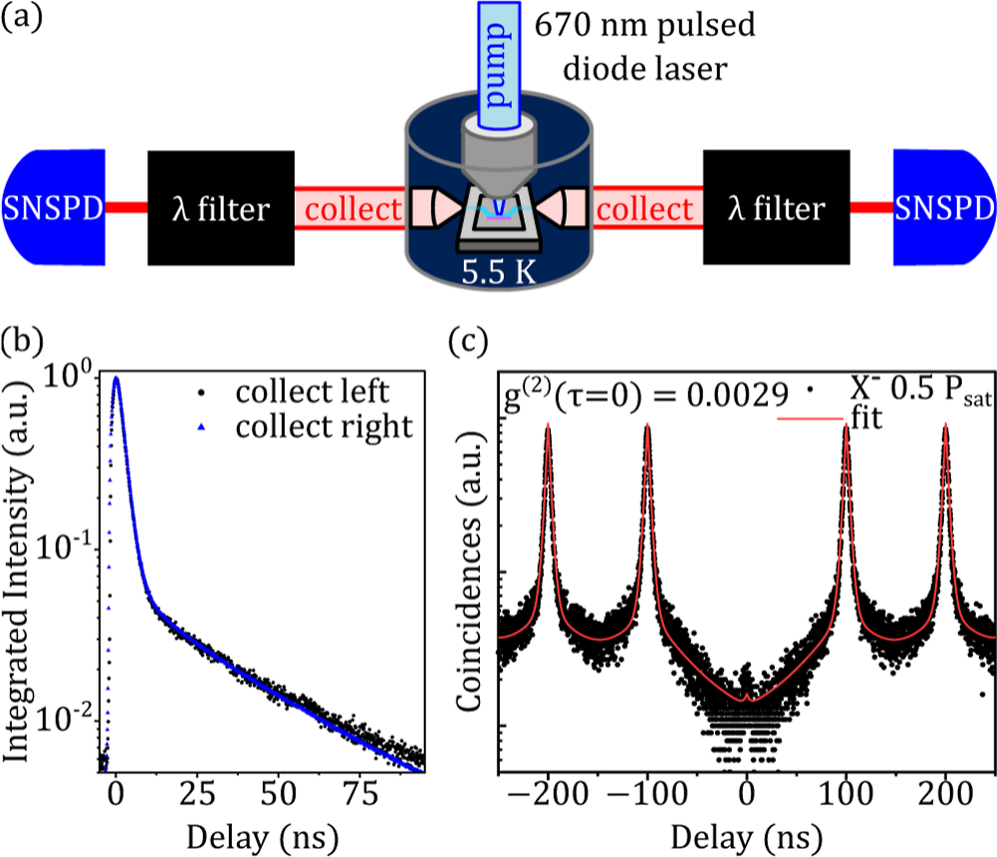
(a) Schematic of bidirectional collection from the gap-coupled device. (b) Comparison of the PL decay of *X* ^–^ emission for collection from the left (black) and right (blue) facets of the chip. (c) Coincidences, *g* ^(2)^(τ), measured using the configuration in (a). For each measurement, the excitation power was 0.5*P*_sat_.

Next, we perform a second-order correlation measurement where collection from both ends of the nanowire replaces the beamsplitter in the HBT setup (compare Figure 4a). The *g* ^(2)^(τ) measured at an excitation power of 0.5*P*_sat_ is shown in Figure 6c. Using eq 2, we obtain *g* ^(2)^(τ = 0) ∼ 0.003, increasing to *g* ^(2)^(τ = 0) ∼ 0.013 at *P*_sat_. The low level of coincidences in the zero delay peak confirms, again, the single photon nature of the emission from the QD.

Using the same configuration, we can also perform cross-correlation measurements by filtering for different complexes at each output. Figure 7a shows the cross-correlation between *XX* and *X* photons where we collect *X* photons from one side of the chip and *XX* photons from the other. With *XX* sent to the “start” detector and *X* to the “stop” detector, we observe strong bunching for τ > 0 consistent with the cascaded *XX* – *X* emission process.^18^

**Figure 7:**
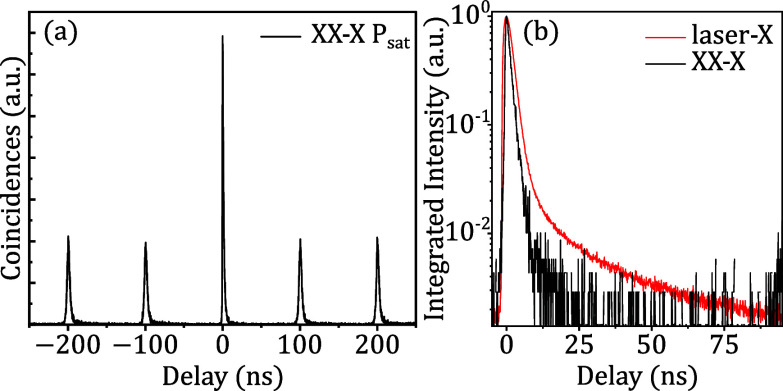
(a) *XX*-*X* cross-correlation measured using bidirectional collection at *P*_sat_. (b) Zoom in showing PL decay of the *X* emission triggered using *XX* detection (black curve) and a synchronization pulse from the laser (red curve).

In Figure 7b, we zoom in on the bunched peak at short delay. This time-resolved PL (black curve) represents the decay of the *X* photon where the start trigger is the detection of an *XX* photon collected from the opposite facet. For comparison, we also show the decay obtained using a synchronization pulse from the laser (red curve), as shown in Figure 4b. Using the laser trigger, we observe a biexponential decay as before with similar extracted parameters: *t*_*b*,*X*_ = 1.765 ± 0.003 ns, *t*_*d*,*X*_ = 16.2 ± 0.3 ns, and *A*_*d*_/*A*_*b*_ = 0.036. Triggering on the *XX* photon, we observe a monoexponential decay with an extracted time constant of *t*_*b*_ = 1.442 ± 0.005 ns, over 300 ps faster. Since detection of the *XX* photon indicates that the system is in the bright *X* state, the slow decay process, associated with a transition from bright to dark via a spin flip at a rate 1/*t*_*d*,*X*_, will never be observed. The 300 ps shorter decay extracted from the cross-correlation trace likely represents a more accurate measure of the radiative lifetime since it is not polluted by excitation and spin-flip processes.

## Summary and Conclusions

We describe a versatile hybrid platform for efficiently generating single photons on-chip based on III–V nanowire QDs integrated with Si_3_N_4_ photonic circuitry. The approach utilizes a nanomanipulator to place individual nanowires on curved Si_3_N_4_ waveguides, providing evanescent coupling of single photons emitted from both ends of the nanowire. We use the bidirectional collection to demonstrate single-photon emission without the need of an external beamsplitter and to perform cross-correlation measurements for accurate determination of the exciton radiative lifetime.

The approach is currently limited by the uncertainty of the dot position within the nanowire (see the Supporting Information) and by the precision with which the nanowire can be placed with respect to the Si_3_N_4_ circuitry. Additionally, the measured nanowires were grown with slight tapers, whereas the device designs are based on perfectly untapered structures. Further growth studies are expected to lead to better control of the nanowire taper^22^ and can also address the growth incubation time,^42^ in part, responsible for the observed variation in dot position. We note that the variation in the dot position within the nanowire is also determined by the variation in the catalyst diameter, see ref 14. Improved nanowire placement can be expected with advances in nanopositioning technologies,^43^ and their automation will address the scalability of device assembly, limited here by the manual nature of the process.

In this study, the sources were operated using above-band excitation, known to cause fluctuations in the charge environment.^44^ Such incoherent excitation introduces excess broadening well above the transform limit^41^ and limits the indistinguishability of the generated photons.^4^ State-of-the-art sources of indistinguishable photons^5–9^ are operated using resonant excitation^45^ and employ microcavity structures^28^ to increase radiative decay rates.^46^ Further development of the current platform thus requires implementation of resonant excitation, already demonstrated in on-chip approaches.^12,30,47,48^ For enhancing decay rates, microcavities based on ring resonators can be used,^49^ a simple extension of the current curved waveguide architecture.

In conclusion, we have proposed architectures for efficiently coupling photons emitted from nanowire QDs to integrated photonic circuits. Such architectures are useful for the study of coherent light–matter interactions with solid-state two-level systems. They will also be required in future quantum information-processing technologies based on the availability of multiple on-chip sources of highly coherent single photons.

## Supplementary Material



## Data Availability

The data that support the findings of this study are available from the corresponding author upon reasonable request.

## Notes

Detailed loss analysis of the device, additional simulations of the dot to waveguide coupling, and a statistical analysis of dot heights within the nanowires (PDF)
