# Supplementary material for: Nanowire Quantum Dot Emitters Evanescently Coupled On-Chip via Curved and Segmented SiN Waveguides
Source: ACS Photonics. 2026 Jul 24;13(15):4185–92. doi: 10.1021/acsphotonics.6c00397 (PMC13450447; doi:10.1021/acsphotonics.6c00397)
Supplement: Supplementary file 1 [file ph6c00397_si_001.pdf]

Nanowire quantum dot emitters evanescently coupled on-chip via curved and segmented SiN waveguides

Edith Yeung,[1,2] Kataryna Sorensen,[1,2] David B. Northeast,[2] Maziyar Milanizadeh,[2] Philip J. Poole,[2] Robin L. Williams,[2] and Dan Dalacu[1,2]

[1] *University of Ottawa, Ottawa, Ontario, Canada, K1N 6N5.*

[2] *National Research Council Canada, Ottawa, Ontario, Canada, K1A 0R6.*

I. ESTIMATED DOT - WAVEGUIDE COUPLING EFFICIENCY

In Table S1 we present estimates of the losses in the optical system. The system loss, obtained from a throughput measurement using a laser, includes off-chip optical components such as the fiber-coupled tunable filter and associated fiber mating sleeves. The waveguide propagation loss, scattering loss at the window openings, and facet coupling loss are obtained from measurements on a cut-back chip fabricated in the same foundry run. Measured propagation losses were -1.5234 dB/cm for the TE mode and -1.3301 B/cm for the TM at 975 nm and the length of the waveguide from gap to edge facet was 25 mm. We assume an ideally positioned, completely untapered nanowire with an embedded dot exactly centered along its length such that 50% of the dot emission is directed towards each half of the segmented waveguide. We further assume a coupling efficiency between the dot and the HE_{11} waveguide mode in the nanowire of $\beta_{\mathrm{HE}_{11}} = 95\%$[1] and a 20% loss from dot emission into acoustic phonon sidebands[2] which is filtered-out.

Source of loss	Transmission (%)	Loss (dB)
Detector efficiency	82	-0.862
Optical system loss	10	-10.0
Propagation loss	91.6 (TE)/ 92.6 (TM)	-0.3809 (TE)/ -0.3325 (TM)
Facet loss	86.9 (TE)/ 68.2 (TM)	-0.6115 (TE)/ -1.6609 (TM)
Window loss	79.3 (TE)/ 82.4 (TM)	-1.006 (TE)/ -0.8413 (TM)
Emission direction	50	-3.0
$\beta_{\mathrm{HE}_{11}}$	95	-0.223
Phonon sidebands	80	-0.969
Total	1.97 (TE)/ 1.62 (TM)	-17.0524 (TE)/ -17.8467 (TM)

TABLE S1. Breakdown of losses for an ideal case with the QD within an untapered nanowire exactly centered in the gap between waveguide segments.

Detected counts for X and X^- were 575 kcps and 330 kcps, respectively, for a total count rate of $I_{meas} = 905$ kcps (i.e. X and X^- are mutually exclusive transitions). Assuming half the emitted photons in the InP nanowire couple to the TE mode of the Si_3N_4 waveguide and half to the TM mode, the total in-fiber count rate is given by $2I_{meas}/(\eta_{\mathrm{TE}} + \eta_{\mathrm{TM}})$ where $\eta_{\mathrm{TE}} = 0.0197$ and $\eta_{\mathrm{TM}} = 0.0162$. Dividing by the laser repetition rate of 80 MHz, we obtain

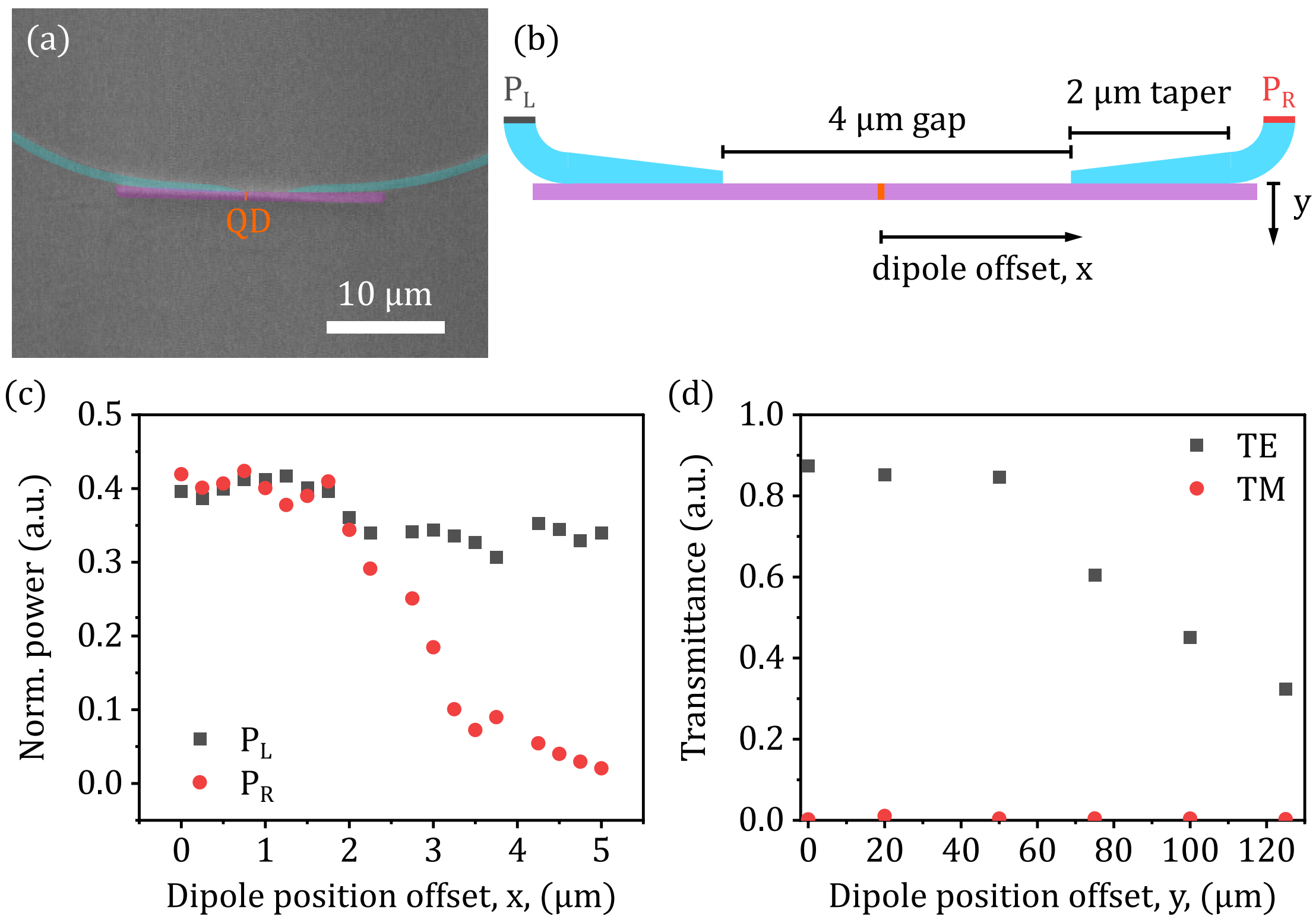

FIG. S1. (a) SEM image of a gap coupler (blue) with a nanowire (purple) where the QD is schematically shown in orange at the nominal position in the middle of the nanowire. (b) Schematic (not to scale) showing the location of ports (P_L and P_R) used for the simulations. (c) Calculated power transfer from the InP nanowire to the P_L and P_R ports as the dipole is shifted from the nominal position, $x = 0$. (d) Calculated transmission of both TE and TM modes through the device as the nanowire is displaced from the Si_3N_4 waveguide from the nominal value of $y = 0$.

an estimated InP nanowire to Si_3N_4 waveguide coupling efficiency of 63% compared to the simulated value of $> 90\%$ from Fig. 2 of the main article.

II. SIMULATIONS FOR POWER TRANSFER DEPENDING ON QD POSITION

The intention of this work's device design is to have the nanowire positioned flush to the Si_3N_4 waveguide tapers and for the quantum dot to be located midway within the gap between the tapers. In this section we investigate the influence of two possible misalignment cases. The first case is axial misalignment of the dipole relative to the gap. The second case looks at instances where the nanowire is increasingly displaced from the Si_3N_4 tapers.

In Fig. S1(a), the dipole position is varied axially in the nanowire, referred to as x in Fig. S1(b), from a centred position ($x = 0\,\mu$m) to a final dipole position just beyond the tapered waveguide section ($x = 5\,\mu$m). The dipole is moved only toward the right-side waveguide. Powers in both waveguides, P_L and P_R, are normalized to the emitted dipole power. Fig. S1(a) shows that, while the dipole is located in the $4\,\mu$m gap section, roughly equal power is delivered to both silicon nitride waveguides. Once the dipole is beside the tapered region of the waveguide, P_R steadily drops as the adjoining waveguide becomes wider. This is in contrast to P_L, which does not drop considerably when the dipole is located beside the right-hand taper. We suspect that this effect is the largest source of count rate asymmetry in our device and suggests a displacement of several microns to account for the $\sim 100\times$ asymmetry observed.

The second series of simulations shows the influence of increasing displacement of the nanowire away from the waveguide, denoted as y in Fig. S1(b). Considered here is only the HE_{11} mode that is aligned to the TE polarization, but the principle will apply equally to the TM mode. Transmittance of mode power into the waveguide is nearly constant until these displacements are greater than 50 nm. At 125 nm, only ~30% of mode power is transmitted into the waveguide. The insensitivity displayed to displacements below 50 nm is due in part to the adiabatic coupling provided by the tapered waveguiding sections.

III. QUANTUM DOT LOCATION WITHIN THE NANOWIRE

To determine the location of the quantum dot within the nanowire, we grew a calibration sample where the growth was stopped right after inserting the dot. An SEM image of an array of these nanowire 'cores' is shown in Fig. S2(a). From measurements of 27 nanowires, we obtain a mean height of $11.1\,\mu$m with and an uncertainty of $\pm 0.7\,\mu$m. This distribution is promising for targeting dot placement to within a couple of microns of the midpoint of the gap, e.g. to simultaneously maximize counts at both P_L and P_R. It also suggests that the studied device had a core height several standard deviations from the mean if the $\sim 100\times$ count rate asymmetry in P_L and P_R is primarily due to the dot displacement within the gap.

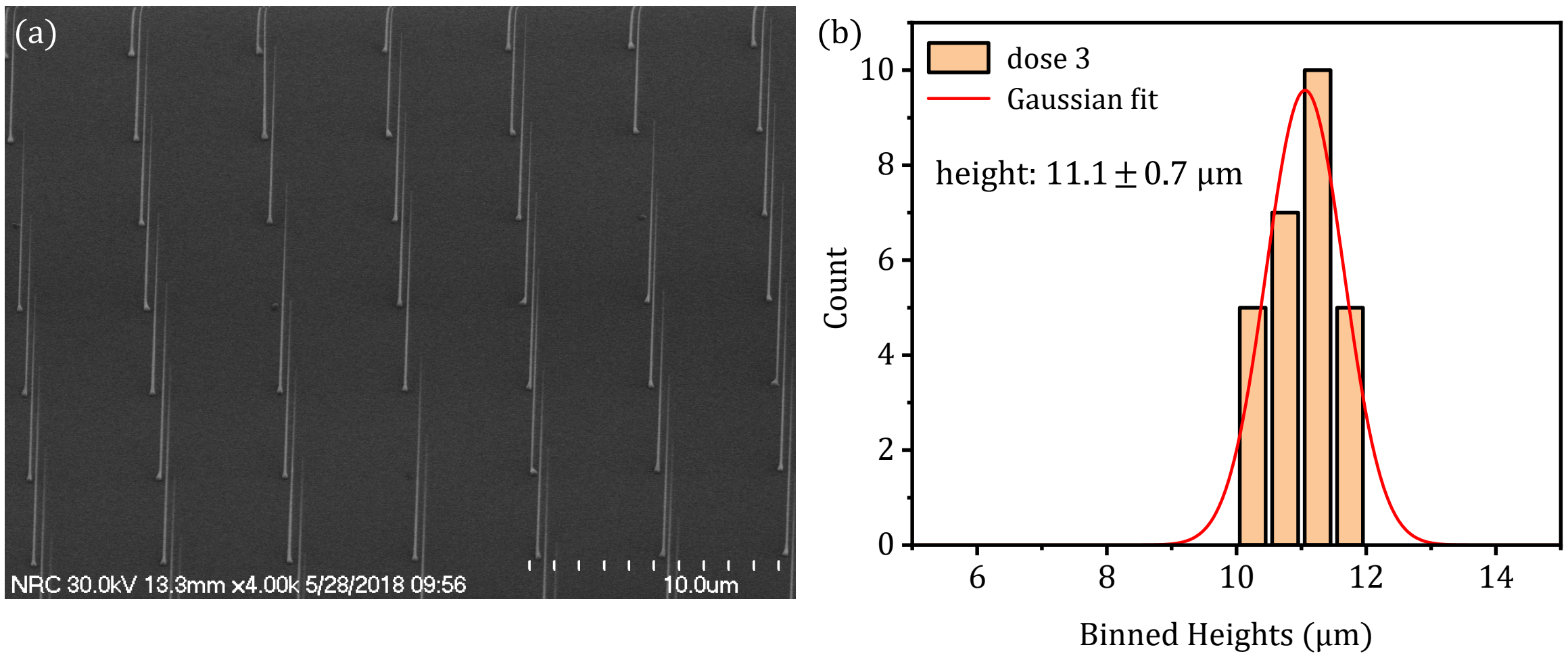

FIG. S2. (a) An SEM image at 45° of an array of nanowire cores defining the position of the quantum dot within the nanowire. (b) Histogram of the heights obtained from measurements of 27 nanowire cores.

---

[1] I. Friedler, C. Sauvan, J. P. Hugonin, P. Lalanne, J. Claudon, and J. M. Gérard, "Solid-state single photon sources: the nanowire antenna," Opt. Exp. **17**, 2095 (2009).

[2] P. Lodahl, S. Mahmoodian, and S. Stobbe, "Interfacing single photons and single quantum dots with photonic nanostructures," Rev. Mod. Phys. **87**, 347 (2015).
